# Correlation between bone mineral density and type 2 diabetes mellitus in elderly men and postmenopausal women

**DOI:** 10.1038/s41598-024-65571-7

**Published:** 2024-07-02

**Authors:** Wei Luo, Xingzhi Li, Yao Zhou, Dan Xu, Yan Qiao

**Affiliations:** 1 Department of Endocrinology, Nanchong Central Hospital, Nanchong, Sichuan China; 2 Department of Hepatobiliary Surgery, Nanchong Central Hospital, Nanchong, Sichuan China; 3 Department of Endocrinology, People’s Hospital of Leshan, Leshan, Sichuan China

**Keywords:** Type 2 diabetes mellitus, Bone mineral density, Insulin resistance, Bilirubin, Osteoporosis, Endocrine system and metabolic diseases, Public health

## Abstract

The relationship between bone mineral density and type 2 diabetes is still controversial. The aim of this study is to investigate the relationship between type 2 diabetes mellitus (T2DM) and bone mineral density (BMD) in elderly men and postmenopausal women. The participants in this study included 692 postmenopausal women and older men aged ≥ 50 years, who were divided into the T2DM group and non-T2DM control group according to whether or not they had T2DM. The data of participants in the two groups were collected from the inpatient medical record system and physical examination center systems, respectively, of the Tertiary Class A Hospital. All data analysis is performed in SPSS Software. Compared with all T2DM group, the BMD and T scores of lumbar spines 1–4 (L1–L4), left femoral neck (LFN) and all left hip joints (LHJ) in the non-T2DM group were significantly lower than those in the T2DM group (P < 0.05), and the probability of major osteoporotic fracture in the next 10 years (PMOF) was significantly higher than that in T2DM group (P < 0.001). However, with the prolongation of the course of T2DM, the BMD significantly decreased, while fracture risk and the prevalence of osteoporosis significantly increased (P < 0.05). We also found that the BMD of L1-4, LFN and LHJ were negatively correlated with homeostatic model assessment-insulin resistance (HOMA-IR) (P = 0.028, P = 0.01 and P = 0.047, respectively). The results also showed that the BMD of LHJ was positively correlated with indirect bilirubin (IBIL) (P = 0.018). Although the BMD was lower in the non-T2DM group than in the T2DM group, the prolongation of the course of T2DM associated with the lower BMD. And the higher prevalence of osteoporosis and fracture risk significantly associated with the prolongation of the course of T2DM. In addition, BMD was significantly associated with insulin resistance (IR) and bilirubin levels in T2DM patients.

**Registration number:** China Clinical Trials Registry: MR-51-23-051741; https://www.medicalresearch.org.cn/search/research/researchView?id=c0e5f868-eca9-4c68-af58-d73460c34028.

## Introduction

Over the past few decades, the prevalence of type 2 diabetes mellitus (T2DM) worldwide has increased dramatically, placing a heavy burden on patients, their families and healthcare system^1^. According to data from China's seventh national population census, the elderly population (≥ 60 years old) accounted for 18.7% (260.4 million) of the total population in 2020. Among them, 30% (78.13 million) had diabetes, of whom more than 95% had T2DM^2^.

Osteoporosis is a systemic metabolic bone disease characterized by reduced bone mass and microstructural deterioration, leading to an increased risk of fractures. The incidence of osteoporosis in postmenopausal women and elderly men increases with age^3^. Other important factors affecting bone fragility in postmenopausal women and elderly men with osteoporosis are the metabolic disorders characteristic of T2DM. These metabolic disorders are associated with disrupted bone homeostasis and a higher risk of fracture^3^.

However, interestingly, one study from Korea showed that lumbar spine and systemic bone mineral density (BMD) in newly diagnosed T2DM patients did not differ from obese control subjects without T2DM after adjusting for potential confounders such as age, sex, body mass index (BMI), etc.; although the BMD of the femoral neck was significantly lower than that of the control^4^. A meta-analysis of 15 observational studies suggested that BMD was significantly higher in patients with diabetes, and, further analysis by meta-regression revealed that individuals with diabetes who were younger and had higher BMI and glycated hemoglobin (HbA1c) were positively correlated with higher BMD^5^. However, one review noted that 13 studies showed a reduction in BMD in patients with T2DM, while another 8 studies found no difference in BMD in patients with T2DM compared with normal controls^6^.

In clinical practice, BMD levels below the osteoporotic threshold and fragile fracture risk assessment tools, such as the World Health Organization fracture risk assessment tool (FRAX), are used to determine when to initiate fracture prevention treatment^7^. However, some studies have reported that these tools underestimate the risk of fracture in patients with diabetes^8^. The reasons for this discrepancy are not entirely clear.

The purpose of this study was to analyze BMD differences between diabetic and non-diabetic patients, fracture risk assessment by FRAX, and identify the associated influencing factors.

## Materials and methods

The participants in this study included 692 postmenopausal women and older men aged ≥ 50 years who were divided into two groups, namely the T2DM group and the non-type 2 diabetes control group based on whether they had T2DM or not. The data of participant subjects in the exposure and control groups were collected between January 2019 and December 2022 from the inpatient medical record and physical examination center systems, respectively, of the Tertiary Class A Hospital. Clinical Ethics Committee of People’s Hospital of Leshan ((LYLL [2023] KY 052)) approved this study. We confirm that all research was performed in accordance with relevant guidelines/regulations. Research involving human research participants have been performed in accordance with the Declaration of Helsinki. The need for informed consent was waived by the ethics committee as the study is retrospective.

In the T2DM group, the patients were divided into three subgroups according to the duration of T2DM: the first group comprised patients with initial T2DM from the onset of T2DM to less than 5 years (A group), the second group comprised patients with a duration of diabetes between 5 and 10 years (B group), the third group comprised diabetic patients with of the disease for more than 10 years (C group). The probability of major osteoporotic fracture in the next 10 years (PMOF) and the probability of hip fracture in the next 10 years (PHF) were calculated using the FRAX tool (https://www.sheffield.ac.uk/FRAX/tool.aspxlang=chs, China model), other FRAX risk factors beyond BMD were obtained by medical records or telephone follow-up. The differences in BMD, fracture risk and related factors were compared among the groups. The instruments we use to measure bone density is: dual-energy X-ray absorptiometry body scan (GE lunar prodigy advance) which is operated by a professional technician.

### Data collection

The subjects were retrospectively analyzed, only postmenopausal women and elderly men aged ≥ 50 years were included in this study. The actual data (clinical and laboratory) collected from the participants included the following: sex, age, BMI, duration of T2DM, fasting blood sugar (FPG), fasting insulin (FINS), systolic blood pressure (SBP), diastolic blood pressure (DBP),alanine aminotransferase (ALT), aspartate aminotransferase (AST),total bilirubin (TBIL), direct bilirubin (DBIL), indirect bilirubin (IBIL), high density lipoprotein (HDL),low density lipoprotein(LDL), total cholesterol (TC), triglycerides (TG), uric acid (UA), HbA1c, thyroid function, bone density examination results. This study was approved by the research ethics committee of Tertiary Class A Hospital.

The following formula was used to calculate homeostatic model assessment-insulin resistance and Insulin secretion index:

$${\text{HOMA}} - {\text{IR}} = \left( {{\text{FINS}}*{\text{FPG}}} \right)/{22}.{5};{\text{ HOMA}} - {\text{IS}} = {2}0*{\text{FINS}}/ \, \left( {{\text{FPG}} - {3}.{5}} \right)$$

**Diagnostic criteria:**

Diagnostic criteria for osteoporosis were the following: dual-energy X-ray absorptiometry (DEXA) body scan. In this study, T scores were measured at the hip, femoral neck, and lumbar spine. Bone density measurements of all patients were performed using the same DEXA body scan machine.

**Table:** 

Diagnostic criteria classification	WHO standard deviation diagnosis
Normal	≥ − 1.0SD
Osteopenia	− 1.0SD ~ − 2.5SD
Osteoporosis	≤ − 2.5SD

Meet the 1999 WHO Type 2 Diabetes Diagnostic Standards:

**Table:** 

Diagnostic criteria	Venous plasma glucose level (mmol/L)
(1) Diabetes symptoms plus random blood sugar	≥ 11.1
or	
(2) FPG	≥ 7
or	
(3) 2hPG	≥ 11.1

Note: If there are no typical symptoms of more than three and one less, it needs to be confirmed by another test. Random blood glucose cannot be used to diagnose IGF or IGT.

### Exclusion criteria

The exclusion criteria for the participants in this study were the following: (1) Patients with type 1 diabetes mellitus (T1DM), patients with diabetes of unknown type, or special types of diabetes; (2) patients with chronic kidney disease, severe renal damage, or end-stage renal disease, or dialysis patients; (3) patients with autoimmune diseases such as rheumatism, systemic lupus erythematosus, rheumatoid arthritis, Sjogren's syndrome*;* (4) To minimize confounding factors, out-of-hospital patients with type 2 diabetes who had long-term insulin use were not included in this study; (5) patients with abnormal thyroid function and abnormal parathyroid function; (6) patients who had malignant tumors within 2 years; and (7) patients who had used glucocorticoids.

### Statistical analysis

The data of this study were analyzed using the SPSS statistical software version 24.0 (IBM Corporation, Armonk, NY, USA). The normally distributed measurement are expressed as the mean ± standard deviation, using a t-test; non-normally distributed data are expressed as the upper and lower quartiles of the median, and the comparison between two independent sample groups was performed using the Mann–Whitney U test. The enumeration data are described by percentage (%), and the ratio between the two groups was tested by a chi-square test. Cox proportional hazards regression analysis was performed to assess the difference in the prevalence of osteoporosis and osteopenia between the T2DM and non-T2DM groups. All variables with P < 0.1 and clinically significant factors were considered using enter method into multivariate analysis. Additionally, multivariate linear regression analysis was performed to assess the influencing factors of BMD in the T2DM group. P < 0.05 indicates that the difference was statistically significant.

### Ethics statement

Clinical Ethics Committee of People’s Hospital of Leshan (LYLL [2023] KY 052); The need for informed consent was waived by the ethics committee as the study is retrospective.

## Results

Our study included 692 postmenopausal women and elderly men aged ≥ 50 years. The data collected from the medical record system included the following: age, sex, BMI, systolic blood pressure (SBP), diastolic blood pressure (DBP), HbA1c, FPG, FINS, high-density lipoprotein cholesterol (HDL-C), low-density lipoprotein cholesterol (LDL-C), total cholesterol (TC), triglyceride (TG), liver function, thyroid function, T value, and BMD (Supplementary Materials: Table S1).

They were divided into the T2DM group (n = 346) and the non-T2DM group (n = 346) according to whether they had T2DM or not. In the T2DM group, patients were further divided into 3 subgroups according to duration of T2DM: 0–5-year T2DM group (A group, n = 170), 5–10-year T2DM group (B group, n = 66), and ≥ 10-year T2DM group (C group, n = 110).

### In all patients

#### General clinical data

There were significant differences in HbA1c, FPG, total bilirubin (TBIL), direct bilirubin (DBIL), indirect bilirubin (IBIL), TG, HDL, LDL, total protein (TP) and albumin (ALB) between the control group (non-T2DM group) and T2DM group (Table 1).

**Table 1:** Comparison of baseline data among all patients (the mean ± standard deviation or median (upper and lower quartile)).

	Non-T2DM	All T2DM	0–5 years T2DM (A)	5–10 years T2DM (B)	≥ 10 years T2DM (C)	P^1^	P^2^	P^3^	P^4^
Age (years)	62 (56, 68)	59 (54, 70)	59 (54, 68)	58.5 (53, 70.5)	63 (55.75, 71)	0.252	0.06	0.228	0.387
BMI (kg/m^2^)	23.88 (21.88, 25.52)	24.21 (22.06, 26.89)	24.52 (22.72, 26.93)	24.04 (21.67, 21.93)	23.51 (21.51, 26.67)	0.052	0.004	0.392	0.82
Gender (male/female)	184/162	203/143	96/74	33/33	55/55	0.168	0.637	0.222	0.122
SBP (mmHg)	130.83 ± 18.19	131 (119, 147.2)	128 (118, 145)	130.5 (119, 148)	135 ± 21	0.108	0.897	0.253	**0.007**
DBP (mmHg)	82 (75, 88)	83.64 ± 12.85	84.13 ± 13.96	85.25 (76.25, 91.25)	82 ± 10	0.053	0.052	0.086	0.697
Osteoporosis (OP/normal)	158/37 (80%)	149/55 (73%)	55/43 (56%)	26/8 (76%)	68/4 (94%)	0.074	**< 0.001**	0.494	**0.004**
Osteopenia (ON/normal)	151/37 (80.3%)	142/55 (73%)	72/43 (63%)	32/8 (80%)	38/4 (90.1%)	0.073	**0.001**	0.557	0.086
HOMA-IR	–	4.24 (2.31, 10.01)	3.61 (2.22, 5.16)	4.02 (2.01, 5.47)	13.76 (3.79, 28.44)	–	–	–	–
HOMA-IS	–	43.1(23.42, 81.57)	47.48 (27.27, 86.49)	50.02 (20.95, 103.49)	43 (27.88, 68.24)	–	–	–	–
HbA1c (%)	5.7 (5.4, 6)	10.93 ± 2.76	10.89 ± 2.79	10.59 ± 3.22	11.2 ± 2.38	** < 0.001**	** < 0.001**	** < 0.001**	** < 0.001**
FPG (mmol/L)	4.91 (4.57, 5.33)	7.9 (6.57, 9.71)	8.01 (6.61, 10.01)	7.58 (6.2, 9.03)	7.87 (6.49, 9.61)	** < 0.001**	** < 0.001**	** < 0.001**	** < 0.001**
FT3 (pmol/L)	4.63 (4.37, 4.83)	4.64 (4.05, 5.15)	4.69 (4.05, 5.1)	4.66 (4.17, 5.37)	10.51 ± 0.88	0.505	0.394	0.188	0.522
FT4 (pmol/L)	15.15 (14.34, 17.37)	15.62 (13.79, 17.41)	15.85 ± 2.79	15.87 ± 2.56	15.48 ± 2.86	0.808	0.879	0.539	0.296
TSH (mIU/L)	2.28 (1.59, 3.13)	2.09 (1.18, 3.45)	2.01 (1.23, 3.54)	2.12 (1.17, 3.34)	2.19 (1.1, 3.47)	0.114	0.117	0.279	0.562
ALT (U/L)	21.1 (15.48, 27.9)	21 (16, 30)	22 (17.5, 32)	22 (15.6, 30)	21 (14, 28.25)	0.07	0.09	0.527	0.81
AST (U/L)	21.1 (18.1, 25.03)	22 (17, 28)	22 (18, 28)	21 (16, 27)	21 (16.75, 28)	0.724	0.217	0.515	0.707
TBIL (umol/L)	12.9 (11, 16.3)	8.2 (5.8, 10.6)	8.7 (6.4, 12)	8.4 (5.55, 10.3)	7.1 (5.18, 9.43)	** < 0.001**	** < 0.001**	** < 0.001**	** < 0.001**
DBIL (umol/L)	4.5 (3.7, 5.48)	2.3 (1.8, 3.2)	2.4 (1.8, 3.5)	2.3 (1.8, 3.1)	2.25 (1.7, 3.1)	** < 0.001**	** < 0.001**	** < 0.001**	** < 0.001**
IBIL (umol/L)	8.6 (7, 10.8)	5.5 (3.68, 7.53)	6.1 (4.08, 7.73)	5.2 (3.6, 7.8)	4.55 (3.1, 6.55)	** < 0.001**	** < 0.001**	** < 0.001**	** < 0.001**
TP(g/L)	75.65(73.1,78.37)	66.4(61.3,71.8)	66.3(61.3,72.6)	66.5(61.95,70.95)	65.8(60.15,71.4)	** < 0.001**	** < 0.001**	** < 0.001**	** < 0.001**
ALB (g/L)	45.35(43.8,46.8)	38.6(34.8,42.65)	38.9(35,4.1)	39(36.35,42.8)	38.2(33.68,41.92)	** < 0.001**	** < 0.001**	** < 0.001**	** < 0.001**
TC (mmol/l)	5.09 (4.43, 5.66)	4.97 (4.18, 6.02)	4.96 (4.32, 6.02)	5.09 ± 1.29	5.06 ± 1.4	0.778	0.896	0.775	0.52
TG (mmol/l)	1.24 (0.91, 1.68)	1.59 (1.14, 2.52)	1.79 (1.17, 3.1)	1.49 (1.18, 2.28)	1.53 (1.11, 2.24)	** < 0.001**	** < 0.001**	**0.002**	** < 0.001**
HDL (mmol/l)	1.49 (1.28, 1.73)	1.1 (0.9, 1.36)	1.04 (0.9, 1.32)	1.13 (0.92, 1.55)	1.14 (0.9, 1.4)	** < 0.001**	** < 0.001**	** < 0.001**	** < 0.001**
LDL (mmol/l)	5.32 ± 1.44	2.91 ± 0.99	2.89 ± 1.03	2.97 ± 1	2.91 ± 0.95	**0.017**	0.062	0.103	0.103
UA (mmol/l)	316.55 (267.95, 366.93)	311 (243, 397)	395.5 (234, 384)	342.79 ± 140.25	339.99 ± 117.76	0.851	0.141	0.59	0.258

*BMI* body mass index, *SBP* systolic blood pressure, *DBP* diastolic blood pressure, *FPG* fasting blood glucose, *ALT* alanine aminotransferase, *AST* aspartate aminotransferase, *TBIL* total bilirubin, *DBIL* direct bilirubin, *IBIL* indirect bilirubin, *HDL* high density lipoprotein, *LDL* low density lipoprotein, *TC* total cholesterol, *TG* triglycerides, *TP* total protein, *ALB* albumin, *UA* uric acid, *FT3* free triiodothyronine, *TSH* thyroid stimulating hormone, *FT4* free thyroxine, *HOMA-IR* insulin resistance index, *HOMA-IS* insulin secretion index, *ON* osteopenia, *OP* osteoporosis, *P1* non-T2DM vs all T2DM, *P2* non-T2DM vs 0-5 years T2DM, *P3* non-T2DM vs 5-10 years T2DM, *P4* non-T2DM vs 5-10yearsT2DM.

Significant values are given in bold.

Also, there were significant differences in HbA1c, FPG, SBP, TBIL, DBIL, IBIL, TG, HDL-C, LDL-C, TP and ALB between the non-T2DM control group and the T2DM group with different duration (Table 1).

#### BMD, T-score, and FRAX-calculated fracture risk

Compared with all T2DM subgroups, the BMD and T scores of lumbar spines 1–4 (L1–4), left femoral neck (LFN) and all left hip joints (LHJ) in the non-T2DM control group were significantly lower than those the in T2DM subgroups (Figs. S1–S6), and the PMOF was significantly higher than that in the T2DM group. There was no significant difference in the PHF between the two groups (Table 2).

**Table 2:** Comparison of BMD, T value and probability of major fracture among all patients (the mean ± standard deviation or median (upper and lower quartile)).

	Non-T2DM	All T2DM	0–5 years T2DM (A)	5–10 years T2DM (B)	≥ 10 years T2DM (C)	P^1^	P^2^	P^3^	P^4^
BMD (g/cm^2^)									
L1–4	0.922 (0.84, 1.02)	0.98 ± 0.22	1.03 ± 0.22	0.95 ± 0.19	0.91 ± 0.21	**0.006**	** < 0.001**	0.567	0.158
LFN	0.752 (0.673, 0.833)	0.81 ± 0.15	0.85 ± 0.16	0.8 ± 0.13	0.76 ± 0.14	** < 0.001**	** < 0.001**	**0.036**	0.77
LHJ	0.827 ± 0.116	0.85 (0.73, 0.96)	0.91 (0.81, 0.99)	0.84 ± 0.17	0.8 (0.68, 0.89)	**0.02**	** < 0.001**	** < 0.001**	**0.031**
T value									
L1–4	− 2.2 (− 2.8, − 1.35)	− 1.8 (− 2.6, − 0.55)	− 1.29 ± 1.77	− 2 (− 2.43, − 1.23)	− 2.2 (− 3.02, − 1.28)	** < 0.001**	** < 0.001**	0.118	0.965
LFN	− 2.1 (− 2.6, − 1.5)	− 1.70 ± 1.12	− 0.75 (− 1.6, − 0.125)	− 1.68 ± 0.88	− 2.11 ± 1.06	** < 0.001**	** < 0.001**	**0.004**	0.371
LHJ	− 1.41 ± 0.933	− 1.2 (− 2.1, − 0.3)	− 0.82 ± 1.23	− 1.16 ± 1.1	− 1.85 (− 2.4, − 0.78)	**0.008**	**0.001**	**0.036**	**0.032**
PMOF%	8.1 (5.5, 11.25)	6.25 (4.2, 10)	5.5 (3.9, 8.78)	6.2 (4.17, 9.12)	7.75 (5.03, 12)	** < 0.001**	** < 0.001**	**0.001**	0.85
PHF%	1.7 (0.8, 3.6)	2.3 (0.7, 5.4)	1.1 (0.4, 2.6)	1.5 (0.5, 3.85)	2.4 (0.8, 5.62)	0.052	** < 0.001**	0.423	0.065

*L1–4* lumbar 1–4, *LFN* left femoral neck, *LHJ* all left hip joints, *PMOF* probability of major osteoporotic fracture in the next 10 years, *PHF* Probability of hip fracture in the next 10 years, *P1* non-T2DM vs all T2DM, *P2* non-T2DM vs 0–5 years T2DM, *P3* non-T2DM vs 5–10 years T2DM, *P4* non-T2DM vs 5–10 years T2DM.

Significant values are given in bold.

Compared with the A group, the BMD and T scores of L1–4, LFN and LHJ in the non-T2DM control group were significantly lower than those in the experimental group, and the PMOF and PHF were significantly higher than those in the experimental group (Table 2) (Figs. S1–S6).

Compared with the B group, the BMD and T scores of LFN and LHJ in the non-T2DM control group were significantly lower than those in the experimental group (Figs. S2, S3, S5, S6), and the PMOF was significantly higher than that in experimental group. Additionally, there were no significant difference in the BMD and T scores of L1–4, and the PHF between the two groups (Table 2).

Compared with the C group, the BMD and T scores of LHJ in the non-T2DM control group were significantly higher than those in the experimental group (Figs. S3 and S6). However, there were no significant differences in the BMD and T scores of L1–4 and LFN between the two groups. Additionally, the PMOF and PHF between the two groups were also not significantly different (Table 2).

### Logistic regression analysis

A comparison between the non-T2DM control group and T2DM group using a binary logistic regression analysis, after adjusting for sex, age, BMI, blood pressure, DBIL, IBIL, TG and HDL-C revealed that there were no significant differences in the morbidity of osteopenia and osteoporosis between the two groups (Table 3).

**Table 3:** OP and ON rate estimated by exposure to T2DM.

	Non-T2DM	All T2DM	Crude OR value (95% confidence interval and P value)	Adjusted OR value (95% confidence interval and P value)
Osteoporosis (OP/normal)	158/37	149/55	OR = 1.576 (0.982, 2.53) P = 0.059	OR = 2.09 (0.987, 4.429) P = 0.054
Osteopenia (ON/normal)	151/37	142/55	OR = 1.581 (0.982, 2.543) P = 0.059	OR = 1.55 (0.842, 2.852) P = 0.159

*BMI* body mass index, *FPG* fasting blood glucose, *DBIL* direct bilirubin, *IBIL* indirect bilirubin, *HDL* high density lipoprotein, *LDL* low density lipoprotein, *TC* total cholesterol, *TG* triglycerides, *Ur* urea, *Cr* glomerular filtration rate, *ON* osteopenia, *OP* osteoporosis.

Model 1: adjusting factors: gender, age, BMI; Model 2: gender, age, BMI, blood pressure, DBIL, IBIL, TG, HDL.

### In patients with different duration of T2DM

#### General clinical data

There was significant difference in HDL-C levels between group A and group B, but no significant differences in the other general clinical index (Table 4).

**Table 4:** Comparison of baseline data among T2DM (the mean ± standard deviation or median (upper and lower quartile)).

	0–5 years T2DM (A)	5–10 years T2DM (B)	≥ 10 years T2DM (C)	P^5^	P^6^	P^7^
Age (year)	59(54, 68)	58.5 (53, 70.5)	63 (55.75, 71)	0.993	0.219	0.054
BMI (kg/m^2^)	24.52 (22.72, 26.93)	24.04 (21.67, 21.93)	23.51 (21.51, 26.67)	0.293	0.889	0.073
Gender (male/female)	96/74	33/33	55/55	0.309	0.877	0.327
SBP (mmHg)	128 (118, 145)	130.5 (119, 148)	135 ± 21	0.335	0.527	0.052
DBP (mmHg)	84.13 ± 13.96	85.25 (76.25, 91.25)	82 ± 10	0.865	0.795	0.371
Osteoporosis (OP/normal)	55/43 (56%)	26/8 (76%)	68/4 (94%)	**0.027**	**0.017**	** < 0.001**
Osteopenia (ON/normal)	72/43 (63%)	32/8 (80%)	38/4 (90.1%)	0.051	0.221	** < 0.001**
HOMA-IR	3.61 (2.22, 5.16)	4.02 (2.01, 5.47)	13.76 (3.79, 28.44)	0.82	** < 0.001**	** < 0.001**
HOMA-IS	47.48 (27.27, 86.49)	50.02 (20.95, 103.49)	43 (27.88, 68.24)	0.944	0.755	0.064
HbA1c (%)	10.89 ± 2.79	10.59 ± 3.22	11.2 ± 2.38	0.469	0.795	0.354
FPG (mmol/L)	8.01 (6.61, 10.01)	7.58 (6.2, 9.03)	7.87 (6.49, 9.61)	0.213	0.532	0.491
FT3 (pmol/L)	4.69 (4.05, 5.1)	4.66 (4.17, 5.37)	10.51 ± 0.88	0.377	0.223	0.539
FT4 (pmol/L)	15.85 ± 2.79	15.87 ± 2.56	15.48 ± 2.86	0.959	0.366	0.286
TSH (mIU/L)	2.01 (1.23, 3.54)	2.12 (1.17, 3.34)	2.19 (1.1, 3.47)	0.975	0.603	0.557
ALT (U/L)	22 (17.5, 32)	22 (15.6, 30)	21 (14, 28.25)	0.648	0.471	0.146
AST (U/L)	22 (18, 28)	21 (16, 27)	21 (16.75, 28)	0.195	0.74	0.247
TBIL (μmol/L)	8.7 (6.4, 12)	8.4 (5.55, 10.3)	7.1 (5.18, 9.43)	0.119	0.087	** < 0.001**
DBIL (μmol/L)	2.4 (1.8, 3.5)	2.3 (1.8, 3.1)	2.25 (1.7, 3.1)	0.296	0.683	0.081
IBIL (μmol/L)	6.1 (4.08, 7.73)	5.2 (3.6, 7.8)	4.55 (3.1, 6.55)	0.241	0.116	** < 0.001**
TP (g/L)	66.3(61.3,72.6)	66.5(61.95,70.95)	65.8(60.15,71.4)	0.931	0.552	0.397
ALB (g/L)	38.9(35,4.1)	39(36.35,42.8)	38.2(33.68,41.92)	0.825	0.365	0.127
TC (mmol/l)	4.96 (4.32, 6.02)	5.09 ± 1.29	5.06 ± 1.4	0.847	0.62	0.678
TG (mmol/l)	1.79 (1.17, 3.1)	1.49 (1.18, 2.28)	1.53 (1.11, 2.24)	0.093	0.888	**0.046**
HDL (mmol/l)	1.04 (0.9, 1.32)	1.13 (0.92, 1.55)	1.14 (0.9, 1.4)	**0.034**	0.687	**0.049**
LDL (mmol/l)	2.89 ± 1.03	2.97 ± 1	2.91 ± 0.95	0.611	0.688	0.902
UA (mmol/l)	395.5 (234, 384)	342.79 ± 140.25	339.99 ± 117.76	0.223	0.84	0.077

*BMI* body mass index, *SBP* systolic blood pressure, *DBP* diastolic blood pressure, *FPG* fasting blood glucose, *ALT* alanine aminotransferase, *AST* aspartate aminotransferase, *TBIL* total bilirubin, *DBIL* direct bilirubin, *IBIL* indirect bilirubin, *HDL* high density lipoprotein, *LDL* low density lipoprotein, *TC* total cholesterol, *TG* triglycerides, *UA* uric acid, *TP* total protein, *ALB* albumin, *FT3* free triiodothyronine, *TSH* thyroid stimulating hormone, *FT4* free thyroxine, *HOMA-IR* insulin resistance index, *HOMA-IS* insulin secretion index, *P5* 0–5 years T2DM vs 5–10 years T2DM, *P6* 5–10 years T2DM vs ≥ 10 years T2DM, *P7* 0–5 years T2DM vs ≥ 10 years T2DM.

Significant values are given in bold.

There were significant differences in HOMA-IR, TBIL, IBIL, TG and HDL-C between group A and group C, but no significant differences in the other general clinical index (Table 4).

There were significant differences in HOMA-IR and creatine (Cr) between group B and group C, but no significant differences in the other general clinical index (Table 4).

#### BMD, T-score, and FRAX-calculated fracture risk

The BMD and T scores of L1–4 and LHJ in group A were significantly higher than those in group B (Figs. S1, S3, S4, and S6). However, there was no significant difference in the BMD and T score of LFN between the two groups. Additionally, the PMOF and PHF between the two groups were also not significantly different (Table 5).

**Table 5:** Comparison of BMD,T value and probability of major fracture among T2DM (the mean ± standard deviation or median (upper and lower quartile)).

	0–5 years T2DM (A)	5–10 years T2DM (B)	≥ 10 yearsT2DM (C)	P^5^	P^6^	P^7^
BMD (g/cm^2^)						
L1–L4	1.03 ± 0.22	0.95 ± 0.19	0.91 ± 0.21	**0.005**	0.264	** < 0.001**
LFN	0.85 ± 0.16	0.8 ± 0.13	0.76 ± 0.14	0.046	0.106	** < 0.001**
LHJ	0.91 (0.81, 0.99)	0.84 ± 0.17	0.8 (0.68, 0.89)	**0.018**	0.15	** < 0.001**
T value						
L1–L4	− 1.29 ± 1.77	− 2 (− 2.43, − 1.23)	− 2.2 (− 3.02, − 1.28)	**0.034**	0.257	** < 0.001**
LFN	− 0.75 (− 1.6, − 0.125)	− 1.68 ± 0.88	− 2.11 ± 1.06	0.107	**0.005**	** < 0.001**
LHJ	− 0.82 ± 1.23	− 1.16 ± 1.1	− 1.85 (− 2.4, − 0.78)	**0.047**	**0.02**	** < 0.001**
PMOF%	5.5 (3.9, 8.78)	6.2 (4.17, 9.12)	7.75 (5.03, 12)	0.378	**0.017**	** < 0.001**
PHF%	1.1 (0.4, 2.6)	1.5 (0.5, 3.85)	2.4 (0.8, 5.62)	0.109	0.054	** < 0.001**

*LFN* left femoral neck, *LHJ* all left hip joints, *PMOF* probability of major osteoporotic fracture in the next 10 years, *PHF* probability of hip fracture in the next 10 years, *P5* 0–5 years T2DM vs 5–10 years T2DM, *P6* 5–10 years T2DM vs ≥ 10 years T2DM, *P7* 0–5 years T2DM vs ≥ 10 years T2DM.

Significant values are given in bold.

The BMD and T scores of L1–4, LFN and LHJ in group A were significantly higher than those in group C (Figs. S1–S6), and the PMOF and PHF in group A were significantly lower than those in group C (Table 5).

The T scores of LFN and LHJ in group B were significantly higher than those in group C (Figs. S2, S3, S5 and S6), and the PMOF in group B was significantly lower than that in group C. There were no significant differences in the BMD of L1–4, LFN and LHJ, and the T scores of L1–4 between the two groups. Additionally, there was also no significant difference in the PHF between the two groups (Table 5).

### Logistic regression analysis and multivariate regression analysis

A comparison between the A group and B group using a binary logistic regression analysis, after adjusting for sex, age, BMI, blood pressure, DBIL, IBIL, TG and HDL-C showed that the morbidity of osteoporosis was significantly higher in B group than that in the A group. The OR and 95% CI values were as follows: OR = 3.465, CI 41.119, 10.729 (Table 6).

**Table 6:** OP and ON rate estimated by exposure to different course of T2DM.

	Osteoporosis (OP/normal)	Crude OR value (95% confidence interval and P value)	Multivariable (model 1)	Multivariable (model 2)	Osteopenia (ON/normal)	Crude OR value (95% confidence interval and P value)	Multivariable (model 1)	Multivariable (model 2)
0–5 years T2DM (A)	55/43	1	1	1	72/43	1	1	1
5–10 years T2DM (B)	26/8	OR = 2.541 (1.046, 6.17) **P = 0.039**	OR = 3.754 (1.287, 10.95) **P = 0.015**	OR = 3.465 (1.119, 10.729) **P = 0.031**	32/8	OR = 2.389 (1.009, 5.656) P = 0.048	OR = 2.06 (0.848, 5.003) P = 0.111	OR = 1.989 (0.799, 4.951) P = 0.139
≥ 10 years T2DM (C)	68/4	OR = 13.291 (4.493, 39.312) **P < 0.001**	OR = 14.655 (4.601, 46.675) **P < 0.001**	OR = 17.327 (4.603, 65.221) **P < 0.001**	38/4	OR = 6.968 (2.02, 24.032) P = 0.002	OR = 6.495 (1.867, 22.593) **P = 0.003**	OR = 9.708 (2.174, 43.349) **P = 0.003**

*BMI* body mass index, *FPG* fasting blood glucose, *DBIL* direct bilirubin, *IBIL* indirect bilirubin, *HDL* high density lipoprotein, *LDL* low density lipoprotein, *TC* total cholesterol, *TG* triglycerides, *HOMA-IR* insulin resistance index, *ON* osteopenia, *OP* osteoporosis.

Model 1: adjusting factors: gender, age, BMI; Model 2: gender, age, BMI, blood pressure, DBIL, IBIL, TG, HDL.

Significant values are given in bold.

A comparison between the A group and C group using a binary logistic regression analysis, after adjusting for sex, age, BMI, DBIL, IBIL, TG and HDL-C revealed that the morbidity of osteopenia and osteoporosis in the C group was significantly higher than that in the A group. The OR and 95% CI values were as follows: OR = 9.708, CI 2.17–43.349 and OR = 17.327, CI 4.603–65.221, respectively (Table 6).

In postmenopausal women and men aged ≥ 50 years with T2DM multiple linear regression analysis was performed using BMD of L1–4, LFN and LHJ as the dependent variables, and the HOMA-IR, HbA1c, FPG, DBIL, IBIL, TG, and HDL-C as the independent variables. The results revealed that the BMD of L1–4, LFN and LHJ were negatively correlated with HOMA-IR (P = 0.028, P = 0.01 and P = 0.047). The results also showed that the BMD of LHJ was positively correlated with IBIL (P = 0.018) (Table 7).

**Table 7:** Multiple linear regression analysis of factors affecting BMD in postmenopausal female and aging male with T2DM.

Variable	Non-standardized coefficient		Standardization factor	T	P	VIF
	B	Standard error	Beta			
BMD L1						
HOMA-IR	− 0.002	0.001	− 0.13	− 2.085	**0.028**	1.06
HbA1c (%)	0.006	0.005	0.072	1.185	0.237	1.135
FPG (mmol/L)	0.002	0.005	0.029	0.488	0.626	1.108
DBIL (μmol/L)	0.006	0.008	0.048	0.749	0.454	1.272
IBIL (μmol/L)	0.004	0.004	0.066	1.032	0.303	1.27
TG (mmol/l)	0.003	0.006	0.025	0.404	0.686	1.137
HDL (mmol/l)	− 0.004	0.004	− 0.062	− 1.076	0.283	1.021
ALB (g/L)	0.005	0.002	0.131	2.126	**0.034**	1.143
BMD LFN						
HOMA-IR	− 0.002	0.001	− 0.158	− 2.582	**0.01**	1.061
HbA1c (%)	− 0.005	0.004	− 0.089	− 1.373	0.171	1.179
FPG (mmol/L)	0.005	0.003	0.099	1.564	0.119	1.136
DBIL (μmol/L)	0.005	0.005	0.061	0.906	0.366	1.273
IBIL (μmol/L)	0.003	0.003	0.072	1.088	0.278	1.254
TG (mmol/l)	− 0.004	0.005	− 0.054	− 0.863	0.389	1.097
HDL (mmol/l)	− 0.002	0.002	− 0.062	− 1.024	0.307	1.023
ALB (g/L)	0.001	0.001	0.059	0.908	0.365	1.153
BMD LHJ						
HOMA-IR	− 0.002	0.001	− 0.122	− 2.001	**0.047**	1.061
HbA1c (%)	− 0.002	0.004	− 0.025	− 0.389	0.698	1.179
FPG (mmol/L)	0.006	0.004	0.113	1.788	0.075	1.136
DBIL (μmol/L)	0.009	0.006	0.103	1.538	0.125	1.273
IBIL (μmol/L)	0.007	0.003	0.158	2.389	**0.018**	1.254
TG (mmol/l)	0.000	0.005	0.005	0.086	0.931	1.097
HDL (mmol/l)	− 0.002	0.002	− 0.049	− 0.816	0.415	1.023
ALB (g/L)	0.002	0.002	0.094	1.475	0.142	1.153

*LFN* left femoral neck, *LHJ* all left hip joints, *HOMA-IR* insulin resistance index, *FPG* fasting blood glucose, *DBIL* direct bilirubin, *IBIL* indirect bilirubin, *HDL* high density lipoprotein, *TG* triglycerides, *ALB* albumin.

Significant values are given in bold.

In postmenopausal women and men aged ≥ 50 years with T2DM multiple linear regression analysis was performed using the PMFO and PHF as the dependent variables, and the HOMA-IR, HbA1c, FPG, DBIL, IBIL, TG and HDL-C as the independent variables. The results showed that there was no significant correlation between the PMFO and PHF and these independent variables (Table S2).

## Discussion

The results of the analyses of 692 postmenopausal women and older men aged > 50 years included in the study showed that BMD was significantly lower in the non-T2DM control group than that in the T2DM group, and the fracture risk was significantly higher in the non-T2DM control group than in the T2DM group. However, the prevalence of osteoporosis and osteopenia was not significantly different between the two groups. Moreover, in the T2DM group, the results showed that the prolongation of the course of T2DM significantly associated with the lower BMD and the higher prevalence of osteoporosis and fracture risk.

Osteoporosis (OP) is an age-related metabolic bone disease, and the acceleration of the aging process has made OP an increasingly major problem affecting the health of a significant proportion of the world population^9^. T2DM has become a worldwide health hazard. The prevalence of T2DM increases significantly with increasing aging of the population^10^. The causes of diabetes and abnormal bone mass are complex and still controversial^11^.

A Mendelian randomization analysis established a causal relationship between T2DM and OP, suggesting a protective effect of T2DM on osteoporosis^12^. Additionally, a meta-analysis of 35 studies showed that patients with T2DM had BMD at the hip and lumbar spine 4–5% higher than non-diabetic patients, which may be due in part to the predominance of overweight and obesity in patients with T2DM^13^. BMD is positively correlated with BMI, which can be considered a physiological phenomenon in which bone mass adapts to large mechanical loads^14^. Other studies have shown that adipose tissue releases a variety of adipokines that are directly or indirectly involved in the regulation of bone remodeling^13,15^. Adipose tissue is the main source of estrogen in postmenopausal female, which increases bone mass by inhibiting osteoclast activity.^16,17^. The mechanism is that peripheral adipose tissue promotes the conversion of and androgens to estradiol and androstenedione to estrone, while leptin and calpain, formed in obesity, suppress the levels of sex hormones and globulin, thereby increasing the levels of free sex hormone level to rise, thus promoting the secretion of sex hormone^16,17^.

However, few studies have reported differences in BMD and its related influencing factors in patients with T2DM of different duration. Remarkably, our study found that the BMD gradually decreased with the extension of the duration of T2DM, and the prevalence of osteoporosis and osteopenia gradually increased with the duration of T2DM. With the prolongation of the course of diabetes, the incidence of diabetic complications also increases. It is considered that diabetic microangiopathy is the risk factor of OP^18,19^. Diabetic microangiopathy (diabetic retinopathy and diabetic peripheral neuropathy) has also been identified as a risk factor for abnormal bone mass^19^. It has been reported that peripheral nervous system in patients with T2DM regulate bone metabolism through the effect of local neurotransmitters on bone cells and neuromodulation of bone vascular supply^18,20^. Because diabetic microangiopathy can cause coordination, balance, and walking problems, physical activity reduction or inactivity is common in diabetic microangiopathy patients, especially in patients with painful diabetic microangiopathy^21^, resulting in decreased bone mass and bone mineral density^22^.

Moreover, we found a significant negative correlation between HOMA-IR and BMD.

The early stage of diabetes is characterized by insulin resistance (IR) and hyperinsulinemia, while the late stage is characterized by β-cell failure, accelerated aging and the development of vascular complications. The direct effect of insulin on bone cells is controversial^13^. Despite evidence from many animal studies^23,24^that insulin stimulates osteoblast proliferation and increases the histomorphometric index of bone formation by two to three-fold, in vitro studies have reported that, insulin signaling in osteoblasts also promotes bone resorption^25^. The concentration of insulin in the bloodstream was also found to be consistent with that of BMD, and showed a positive correlation^26,27^. Similarly, extensive clinical studies on hyperinsulinemia, including polycystic ovary syndrome and lipodystrophy, have consistently found high BMD^28,29^. A study by Liefde et al*.* reported finding higher BMD only in patients with newly diagnosed T2DM and impaired glucose tolerance compared with normal glucose tolerance^30^. However, the long-standing T2DM patients were found to have a significantly higher fracture risk than the healthy controls, while the patients with impaired glucose tolerance had a lower fracture risk than the healthy controls, indicating that bone fragility, progressive β-cell dysfunction and IR are associated with the evolution of the disease^30^. In this study, we found that the risk of fracture was higher in the long-term T2DM group than in the 0–5 years T2DM group, it is possible that bone deterioration in these patients resulted in increased fractures^31^ (Table 5). Although the risk of fracture in the next 10 years was not higher in the T2DM group than in the non-T2DM group. This may be due to the fracture risk assessment tool (FRAX), widely used to estimate 10-year absolute fracture risk, which has been shown to underestimate the risk of hip and major osteoporotic fractures in patients with T2DM^32^. These results are partly influenced by the higher BMD in patients with T2DM^5^.

BMD and fracture risk depend on the stage of T2DM development. Early T2DM is dominated by protective factors, including obesity and hyperinsulinemia. However, as the disease progresses, risk factors, including the development of acute gastroenteritis and other effects of reactive oxygen species, as well as the development of chronic complications^33–36^. These factors will eventually lead to the onset of diabetes and the increase of BMD at the initial stage. Subsequently, depending on the different time intervals of IR, blood glucose control, and individual genetic susceptibility, the onset of aging is accelerated when bone loss accelerates and bone fragility ensues^13,37^. Additional, elderly patients with long-term diabetes may have poorer nutritional status as they age^38^, affecting bone metabolism^39^.

In this study, a significant positive correlation was found between BMD and IBIL. The longer the duration of the disease, the lower the levels of various anti-inflammatory factors, such as bilirubin, which are beneficial to the body, mainly due to the decrease of the function of β-islet cells and the level of bilirubin with the prolongation of the duration of the disease that ultimately exacerbates the body's chronic low-grade inflammation. The conjugated double bonds in the structure of bilirubin can trap oxygen free radicals, and its activated hydrogen atom can scavenge oxygen free radicals and protect cells from oxidative stress damage caused by hydrogen peroxide, thereby reducing the body's chronic inflammatory state^40,41^. Recent studies have found that under physiological conditions bilirubin is an important antioxidant and anti-inflammatory factor in the body^42^. It has also been shown that the proper concentration of bilirubin can protect the function of the islets of Langerhans as well as patients from diabetic macroangiopathy and microangiopathy^43^. Pro-inflammatory conditions and oxidative stress have a strong effect on bone metabolism^37,44^. Microvascular disease and impaired association of vascular smooth muscle cells with bone determine changes in bone mass and microstructure^37,44^. Therefore, bilirubin may play an important role in bone metabolism and BMD in T2DM patients.

In this study, we showed the differences in BMD and fracture risk between the T2DM group and the non T2DM group. Additionally, this is the study to investigate the differences in BMD and fracture risk patients with different T2DM durations, as well as the relevant influencing factors.

However, this study has certain limitations, including the following: it is an observational study and thus can only answer related relationships, and not causal relationships. Due to the paucity of clinical data and since this is a retrospective study, other parameters associated with T2DM and BMD, for example, complications of diabetes, socioeconomic data (such as calcium intake, exercise habits) and the type and dosage of glucose-lowering drugs were not available for all patients. Finally, all participants included in the experimental groups of this study were T2DM inpatients, and most of the patients were admitted due to poor glycemic control. This may explain the lack of significant differences in HbA1c levels between T2DM patients with different disease durations. Although we did not perform a sex subgroup analysis, with no difference in sex composition between the non-T2DM control group and the T2DM group (P > 0.05).

In conclusion, our study found that BMD was lower in the non-T2DM control group than in the T2DM group, but the prolongation of the course of T2DM associated with the lower BMD. And the higher prevalence of osteoporosis and fracture risk significantly associated with the prolongation of the course of T2DM. BMD was significantly correlated with IR and IBIL in T2DM. However, the mechanism of this relationship is not fully understood, and more comprehensive large-scale clinical studies, especially prospective studies, need to be conducted.

### Supplementary Information

Supplementary Figures.

Supplementary Table 1.

Supplementary Table 2.

## Data Availability

The datasets used and/or analysed during the current study available from the corresponding author on reasonable request.
