# Supplementary material for: Correlation between bone mineral density and type 2 diabetes mellitus in elderly men and postmenopausal women
Source: Sci Rep. 2024 Jul 2;14:15078. doi: 10.1038/s41598-024-65571-7 (PMC11219895; doi:10.1038/s41598-024-65571-7)
Supplement: Supplementary file 1 — Supplementary Figures. [file 41598_2024_65571_MOESM1_ESM.docx]

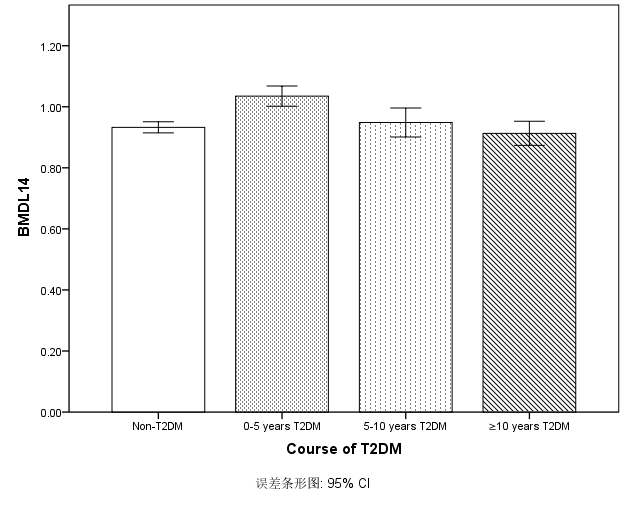


Figure S1 Comparison of BMD of lumbar vertebra (L1-L4) in different course of type 2 diabetes mellitus.


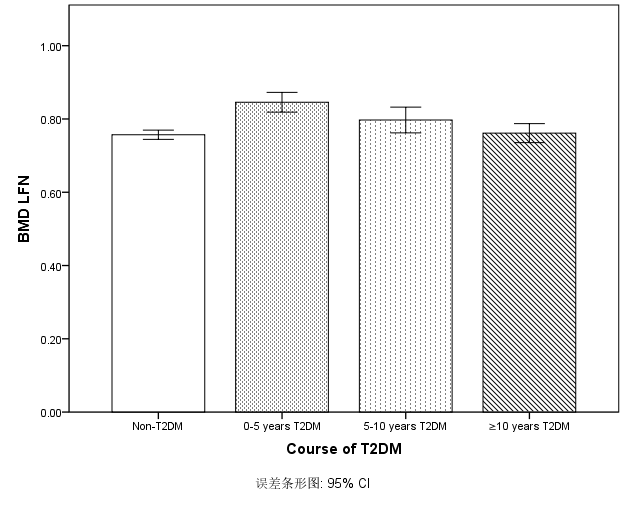


Figure S2 Comparison of BMD of left femoral neck in different course of type 2 diabetes mellitus.


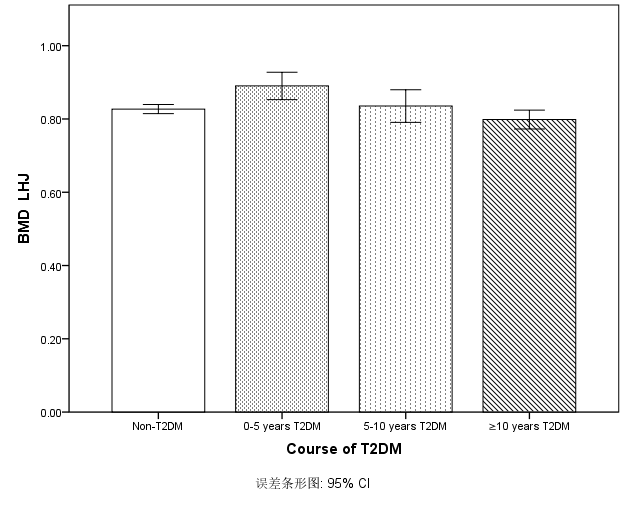


Figure S3 Comparison of BMD of all left hip joints in different course of type 2 diabetes mellitus.


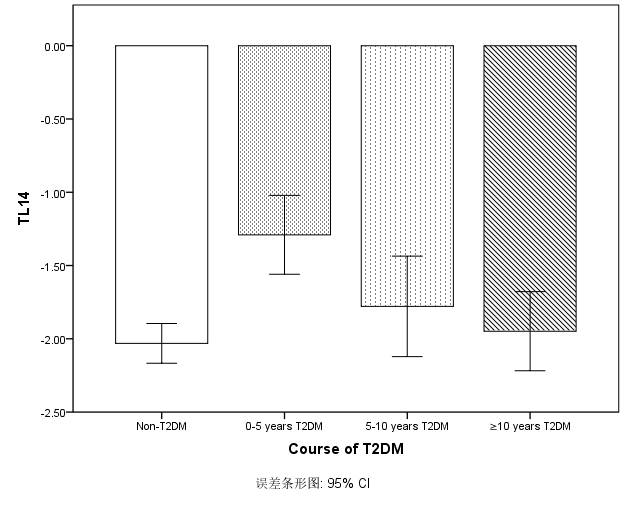


Figure S4 Comparison of T value of lumbar vertebra (L1-L4) in different course of type 2 diabetes mellitus.


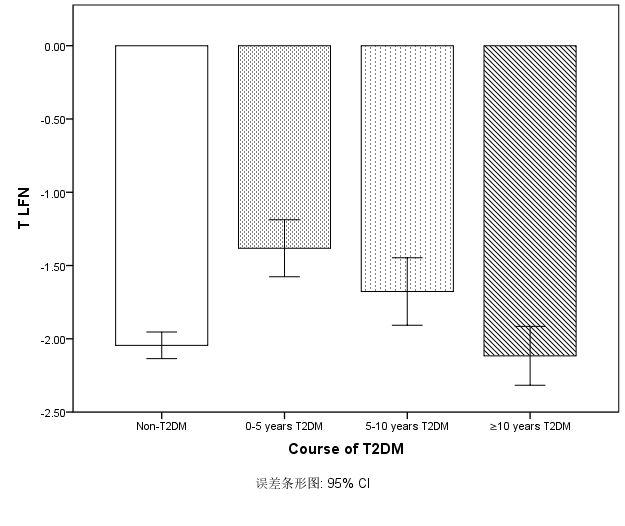


Figure S5 Comparison of T value of left femoral neck in different course of type 2 diabetes mellitus.


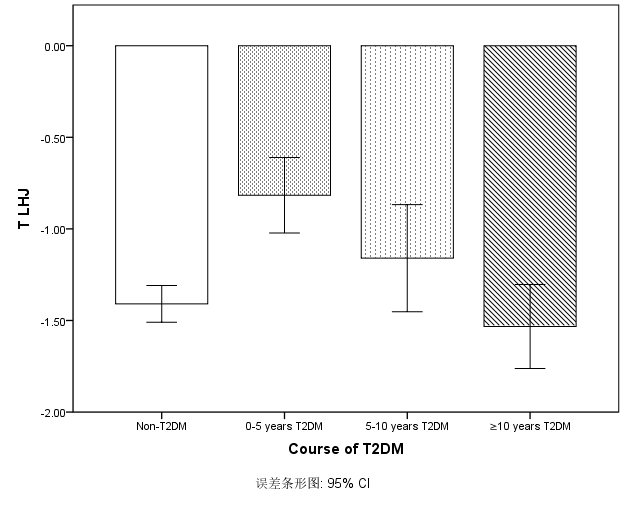


Figure S6 Comparison of T value of all left hip joints in different course of type 2 diabetes mellitus.
